# Genetic Diversity and Structure Analysis of *Percocypris pingi* (Cypriniformes: Cyprinidae): Implications for Conservation and Hatchery Release in the Yalong River

**DOI:** 10.1371/journal.pone.0166769

**Published:** 2016-12-02

**Authors:** Xiaoyan Li, Yuanping Deng, Kun Yang, Weixiong Gan, Rukui Zeng, Longjun Deng, Zhaobin Song

**Affiliations:** 1 Sichuan Key Laboratory of Conservation Biology on Endangered Wildlife, College of Life Sciences, Sichuan University, Chengdu, China; 2 Yalong River Hydropower Development Company, Ltd., Chengdu, China; 3 Key Laboratory of Bio-Resources and Eco-Environment of Ministry of Education, College of Life Sciences, Sichuan University, Chengdu, China; Embrapa, BRAZIL

## Abstract

*Percocypris pingi* is a near threatened cyprinid species, which has suffered a dramatic decline due to anthropogenic factors. As one response to this decline, hatchery release for *P*. *pingi* has been conducted in the lower reaches of the Yalong River since 2012. To understand the conservation status of this species and the potential impact of the release of hatchery-reared fish, we studied the genetic diversity and population structure of wild and hatchery populations of *P*. *pingi*. Two hatchery populations (Jinping [JPH] and Ya’an [YAH]) and two wild populations (Muli [MLW] and Woluo [WLW]) of *P*. *pingi* were analyzed based on microsatellite markers and the mitochondrial DNA control region. The results showed that *P*. *pingi* possesses moderate levels of genetic diversity, with observed heterozygosities ranging from 0.657 to 0.770 and nucleotide diversities ranging from 0.00212 to 0.00491. Our results also suggested WLW harbors considerable proportions of genetic diversity in this species and serves as a refuge for *P*. *pingi* during anthropogenic disturbance, thus playing an important role for the conservation of *P*. *pingi* populations. Microsatellite and mitochondrial markers both indicated close genetic relationships between YAH and MLW, JPH and WLW, respectively. The results to some extent reflected the geographical provenances for original broodstocks of the two hatchery populations, which provide some practical guidance for hatchery release of *P*. *pingi*. The existence of remarkable genetic divergence distributed along limited geographical range (approximately 10 kilometers) suggests the two wild populations should be regarded at least as two distinct evolutionary significant units (ESUs) and management units (MUs). Considering reduced intra-population genetic variation in hatchery population for release and significant genetic compositions of the two hatchery populations, some appropriate breeding strategies were proposed to benefit conservation of *P*. *pingi*.

## Introduction

*Percocypris pingi* (Tchang) (Cypriniformes: Cyprinidae) is an important commercial fish [1] distributed in the upper reaches of the Yangtze River and its tributaries [2,3,4], mainly in the Qingyi, Dadu, Yalong and Jinsha rivers. *Percocypris pingi* prefers lotic habitats and spawns several thousand eggs in these lotic habitats once each spring [1]. Because of large size and delicious taste, the fish has been strongly targeted and overexploited by commercial fishermen. In addition, other anthropogenic interferences, such as habitat deterioration and water pollution, have led to further decline of *P*. *pingi* populations [3,5]. *Percocypris pingi* was once widely distributed in the main channel and tributaries of the Yalong River, the largest branch of the Jinsha River, which runs through the provinces of Qinghai and Sichuan in western China. Due to the construction of a number of series of hydropower stations and overexploitation, *P*. *pingi* populations in this river have decreased dramatically in recent years, and its distribution range has been drastically reduced [6,7]. It now occurs mainly in the lower reaches of the Yalong River, the Muli River (a tributary of the Yalong) and the Woluo River (a tributary of the Muli). Also, Wild resources of *P*. *pingi* in the Qingyi and Dadu rivers were very limited based on our visiting investigation. The continuously deteriorating conservation status of *P*. *pingi* has led to its classification as a near threatened species for conservation in the IUCN Red List [8], and its listing as a protected wild animal in Sichuan Province. In May 2015, *P*. *pingi* was classified as Endangered (EN) in the Red List of China’s Vertebrates [9].

Specific conservation strategies, and additional studies on population genetics of *P*. *pingi* that could provide crucial information relevant for conservation are urgently needed. Conservation management plans with no prior knowledge of the genetic background could result in disturbance to the population structure with adverse effects on the gene pools of wild populations [10]. However, little knowledge of population genetics is available for *P*. *pingi*. Previous studies of *P*. *pingi* focused more on morphology, anatomy, nutriology, and artificial propagation with a few studies involved with molecular genetic issues [11,12,13]. Only a single study conducted by Yue et al. [14] investigated the genetic diversity of one *P*. *pingi* population from the Mianning section of the Yalong River, using mitochondrial control region dataset. The genetic diversity and population structure of *P*. *pingi* hasn’t been carefully characterized until now, which has posed a major hindrance to conservation and management of this species.

**Fig 1:**
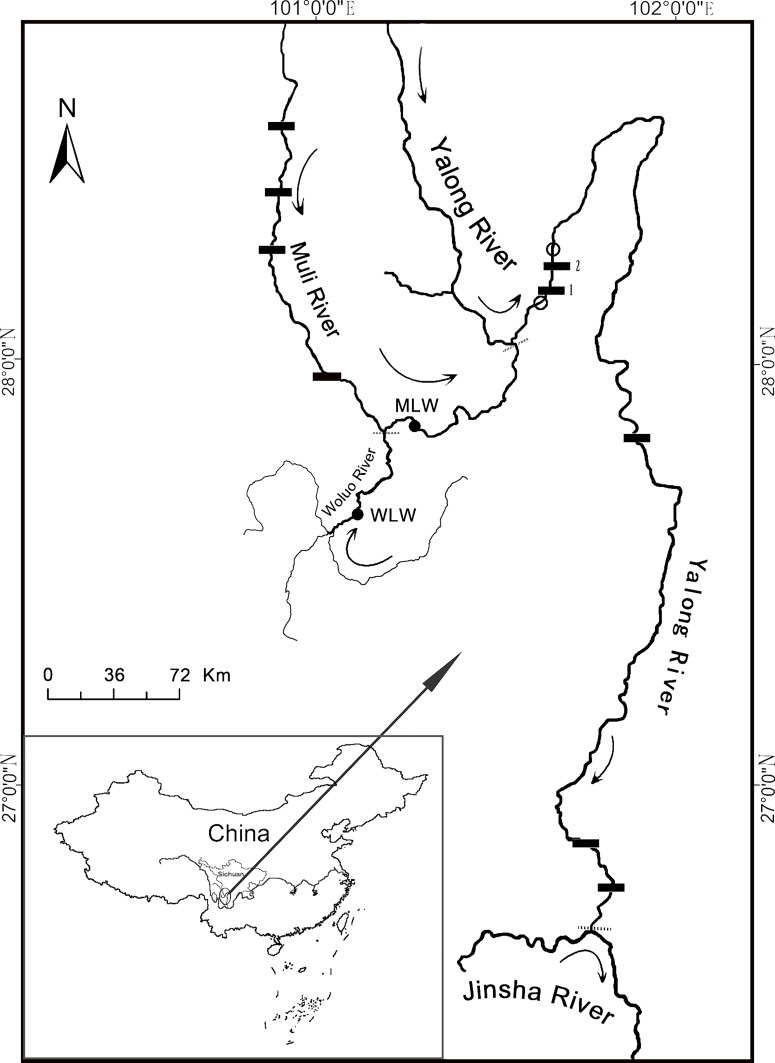
The sampling locations of wild *P*. *pingi* in this study. Curved arrows, flow direction of the rivers; solid circles, sample sites; rectangles, hydropower dams (1 = the Jinping I dam, located approximately 17.5 km downstream from the Yalong River and Muli River confluence; 2 = the Jinping II dam, located 7.5 km downstream from the Jinping I dam); hollow circles, release sites for hatchery-reared *P*. *pingi*; dotted lines, boundaries between different river flows. Base maps are available from NFGIS (national fundamental geographic information system http://nfgis.nsdi.gov.cn/).

Release of hatchery-reared individuals into the wild has been regarded as an efficient conservation strategy for natural fish populations, which has been widely practiced for many fishes and helped to increase local populations of some species such as *Mugil cephalus* and *Rutilus frisii kutum* [15,16]. Artificial propagation of *P*. *pingi* has been conducted in several institutions, including the Fish Reproduction Station of Jinping-Guandi and the Zhougonghe Yayu Company in Ya’an. In order to restore populations of the species in the Yalong River, artificially propagated individuals have been released into the river by the Fish Reproduction Station of Jinping-Guandi beginning in 2012. In 2012, hatchery-reared *P*. *pingi* (around 5 cm in total length) were released into downstream of dam of the Jinping II Hydropower Station, the second dam downstream from the Yalong River and Muli River confluence; in 2013, hatchery-reared *P*. *pingi* (had body length 4.8–9.2 cm) were released just above of the Jinping I dam and below the Jinping II dam on the same river stretch (see the release sites identified by the hollow circles in Fig 1). However, these were carried out without regard to detail archives of the broodstocks of the species in the hatcheries, which could have long-term unintended negative consequences for wild populations.

The genetic quality of hatchery-reared juveniles may limit the effectiveness of release activities. Hatchery populations are commonly considered to show a reduced genetic variability caused mainly by the inbreeding depression associated with small effective population sizes and limited broodstocks [17,18]. Releasing hatchery-reared fish with reduced genetic variability to natural areas may possibly reduce genetic diversity of wild populations, the consequences of which can reduce productivity and fitness [19]. Such deleterious genetic impacts of captive breeding and hatchery release on wild population restoration have been observed in hatchery release activities for several species [20,21,22]. Additionally, when hatchery populations are genetically divergent from wild populations, severe reductions in fitness and changes in genetic structure of the wild populations may occur [23], as in the case of salmonids and largemouth bass [24,25]. Therefore, a clear picture of genetic diversity and population structure of hatchery-reared compared to wild populations is strongly recommended before carrying out conservation strategies.

Genetic assessment within and between populations can be implemented by either DNA or protein-based methods. Among the several marker systems, microsatellite markers are the most currently used due to their high variability, codominance, Mendelian inheritance, and simple evolutionary models [26,27]. The mitochondrial DNA control region was demonstrated exhibits elevated levels of variation relative to coding sequences [28], which consequently supplements the analysis of nuclear markers. Some authors considered that the consolidation of mtDNA and microsatellite markers would greatly improve the overall detectability of genetic diversity [29].

In the present study, we used a combination of microsatellite markers and mtDNA control region to evaluate the genetic diversity and population structure of hatchery and wild populations of *P*. *pingi*. The genetic information obtained by this study could help to establish appropriate breeding strategies and provide essential data for genetic management during conservation and hatchery release of this species.

## Materials and methods

### Sample collection and DNA extraction

The wild individuals were caught from the Muli River in September and December, 2013 (MLW: 101°16’27.71”E, 27° 50’25.42”N, 40 individuals, body length 16.0–47.0 cm) and Woluo River in October, 2013 (WLW: 101°7’22.13”E, 27°37’8.57”N, 65 individuals, body length 8.3–31.5 cm) (Fig 1). To ensure our wild samples were not artificially propagated individuals, we have the following two considerations: (1) Sample locations (the Muli and Woluo rivers, see Fig 1), both upstream from the Jinping I dam on the Yalong River were chosen to ensure our wild *P*. *pingi* were not intermixed with hatchery-released ones. Since the hatchery *P*. *pingi* released in 2012 dispersed into downstream of the Jinping II dam (see details in Introduction). Its upstream passage into the Yalong River and its tributaries was blocked by this dam (with height of 34meters) as well as the Jinping I dam (a high arch dam with height of 305 meters) [30]. The Muli and Woluo rivers thus became two important tributaries in lower reaches of the Yalong River where wild *P*. *pingi* populations didn’t be perturbed by the hatchery release that was done in 2012 below the Jinping II dam on the Yalong River. (2) Most of the wild fish were collected before the hatchery release in 2013 (hatchery release of this year conducted on October 30, 2013) to ensure that the “wild individuals” collected were not likely to include any hatchery-reared fish. However, due to the small number of *P*. *pingi* sampled in the Muli River, additional samples (had body length more than 22 cm, which are much larger than released fish) were collected in December, 2013 (after the release). The released ones of this year all had body length 4.8–9.2 cm and couldn’t grow to the size as that we collected even in December of this year.

The sample locations were public and no specific permission was required to access these locations. Since *P*. *pingi* was a listed as a protected species in Sichuan Province, we sampled *P*. *pingi* with approval of the Sichuan Municipal Bureau of Aquatic Products (S3 Fig) and conducted field studies according to the ethical standards approved by the Sichuan University Medical Ethics Committee. We anesthetized the fish immediately with MS-222 at a concentration of 100 mg·L^-1^ [31], measured body size to the nearest millimeter and mass to the nearest gram, sampled tail fin clips (then preserved in 95% ethanol for DNA extraction), and released the fish collected on-site following recovery from anesthesia.

The hatchery individuals were collected from the Fish Reproduction Station of Jinping-Guandi (JPH, 41 individuals, body length 6.0–9.2 cm) and the Ya’an Zhougonghe Yayu Company (YAH, 33 individuals, body length 11.8–17.8 cm) from 2013 to 2014. These samples were preserved in 95% ethanol for DNA extraction.

Total DNA was extracted from fin tissues by TIANamp marine animals DNA kit (Tiangen, China).

### Microsatellite amplification and genotyping

Twelve tetranucleotide microsatellite markers were used for analyses: Seven (PM49, PM32, PM25, PM23, PM20, PM19 and PM15) from Deng et al. [13] and another five (PM10, PM17, PM16, PM08, PM40) from Deng [32] (see S1 Table for details on the loci). Each forward primer of the loci was labeled by fluorescent dyes–FAM, TAMRA or HEX–for fragment visualization. A polymerase chain reaction (PCR) was performed using a standard PCR protocol described in Deng et al. [13].

All amplified products were initially examined on 1.5% agarose gels stained with GelGreen at 90 V for 14 min and were then sent to Beijing Microread Genetics Co., Ltd. (Beijing, China) for genotyping and fragment analysis. We then read the genotyping data manually from chromatograms. MICRO-CHECKER 2.2.3 [33] was used to test the data for genotyping errors due to stutter peaks and null alleles.

### MtDNA amplification, sequencing and alignment

The mtDNA control regions were obtained using the primer sets DL1 (ACCCCTGGCTCCCAAAGC) and DH2 (ATCTTAGCATCTTCAGTG) [34]. The PCR was run in 25μL volumes containing 3.0 μL 10 × PCR buffer (TaKaRa, Japan), 0.7μL deoxyribonucleoside triphosphates (2.5 mM), 0.3 μL each forward and reverse primers (10 μM), 0.3 μL Taq polymerase (5 U/ μL), approximately 200ng genomic DNA and 19.40 μL of double-distilled H_2_O. The PCR was programmed as follows: an initial denaturation at 94°C for 4min; 35 cycles of a denaturation at 92°C for 30s; an annealing at 62°C for 1 min; an elongation at 72°C for 1 min; and a final extension at 72°C for 10 min.

After amplification, the PCR products were purified using a universal DNA purification Kit DP214-02 (Tiangen, China) and sequenced directly on an ABI 3730XL automatic sequencer by the Invitrogen Corporation (Shanghai, China). The sequences were then proofread and assembled with the DNA analysis package DNASTAR Lasergene Seqman (DNAStar Inc., Madison, WI). All sequences were aligned using the Clustal W application in MEGA 7.0.14 [35] and refined manually. Sequences of the different haplotypes were deposited in GenBank (KX590528 to KX590556).

### Data analysis

#### Genetic diversity

The microsatellite genetic diversity estimation, the tests for Hardy–Weinberg equilibrium (HWE), and linkage disequilibrium among loci followed Deng et al. [13]. We evaluated the mean number of alleles per locus and per population (*A*), mean allelic richness across all loci per locus and per population (*A*_*i*_), the polymorphic information content (PIC), the observed heterozygosity (*H*_*o*_), and expected heterozygosity under random chromosome segregation (*H*_*e*_(*c*_*e*_)) and under some level of chromatid segregation (*H*_*e*_(*c*_*d*_)). Allele frequencies and inbreeding coefficients (*F*_*IS*_) per locus and per population were calculated in Fstat v2.9.3.2 [36]. Further, rare and private alleles were calculated based on allele frequencies. P-values were calculated and corrected for multiple tests using the Bonferroni method [37].

The number of haplotypes (h), haplotype diversity (*H*_*d*_) and nucleotide diversity (*π*) of mtDNA control region were calculated using DnaSP v.5.10.01 [38]. Haplotype frequency was determined using Arlequin 3.5.1.3 [39].

#### Genetic differentiation and population structure

AMOVA was run for estimating the partitioning of genetic variation within and between populations (or groups) for the two markers in Arlequin. Estimates of significance were obtained by 1 × 10^4^ permutations. The genetic divergence among populations was calculated as *F*_*ST*_ for microsatellite and *Φ*_*ST*_ for mitochondrial data with the same software. Then the neighbor-joining dendrograms based on *F*_*ST*_ and *Φ*_*ST*_ were built by the MEGA program.

The patterns of the population structure were investigated using the model-based Bayesian clustering procedure in STRUCTURE v.2.3.3 [40]. We tested the admixture model with a set of K values (number of clusters) ranging from 1 to 8. Ten independent runs of each K were performed with 1 × 10^6^ repetition and 1 × 10^5^ burn-in period.

Moreover, the median-joining haplotype network from mtDNA data was established in Network 4.6.1.1 (Fluxus Technology Ltd http://www.fluxus-engineering.com).

#### Demographic analyses

To test for past population dynamics of the four *P*. *pingi* populations, Tajima's D [41] and Fu's FS [42] parameters were calculated in Arlequin. The Bayesian Skyline analysis was implemented in BEAST v.1.8.3 [43] and visualized in TRACER v.1.6 [44]. Final analyses were run for 1 × 10^6^ generations, sampling every 100th generation including a burn-in of 1 × 10^5^ generations. In addition, the bottleneck signature for microsatellite marker was obtained from Wilcoxon’s signed-rank and mode-shift tests in BOTTLENECK v.1.2.02 [45]. A Two-Phase Model (TPM with 90% SMM and 10% IAM) was estimated, considering that it is more realistic than tests for bottlenecks using Wilcoxon’s test [46].

#### Equilibrium testing and historical gene flow

To detect the relative probability of two models of migration-drift equilibrium versus pure drift for the two wild populations, program 2MOD v.0.2 [47] was used for analyzing microsatellite data. The first model assumes that the gene frequencies within populations are caused by a balance between genetic drift and gene flow, and the second assumes differentiation among populations is caused by isolation of the ancestral population with no subsequent gene flow [48]. We evaluated these two models in separate runs (0 indicates drift model and 1 indicates migration–drift model, which are two alternative models when running program 2MOD v.0.2, indicating which model is currently being used), each with 1 × 10^5^ iterations. The first 10% of iterations were discarded as burn-in.

To calculate the migration rates between two wild populations, the program IMa2 [49] was used by analyzing mitochondrial data based on the IM model. We ran 40 chains with dynamic heating (-ha 0.975 -hb 0.75) and uniform priors (-q 20 -m 0.5 -t 8), collecting 20,000 from 100,000 generated topologies (The first 1,000 were discarded as a conservative burn-in).

## Results

### Microsatellite polymorphism

Twelve microsatellites were used to screen 179 individuals and generated 172 alleles. MICRO-CHECKER did not find any genotyping errors due to shutting or null alleles. Accordingly, the total numbers of alleles varied from 9 (PM40) to 16 (PM17) *(mean =* 13) per locus and from 110 (MLW) to 141 (WLW) per population across all loci (S2 Table). Of the 172 microsatellite alleles, 54 were private alleles: 20 in MLW, 15 in WLW, 10 in JPH, and 9 in YAH. The highest number of alleles (*A =* 11.75), mean PIC values (*PIC =* 0.801), and expected heterozygosity under random chromosomal segregation (*H*_*e*_(*c*_*e*_) = 0.820) and under random chromatid segregation (*H*_*e*_(*c*_*d*_) *=* 0.765) were observed in WLW. In comparison, the MLW displayed the lowest number of alleles, PIC values, observed heterozygosity, and expected heterozygosity under two assumptions (Table 1).

**Table 1:** Genetic diversity of four *P*. *pingi* populations inferred as from two kinds of markers.

Populations			Microsatellite						mtDNA				
	*N*	*A*	*A*_*i*_	*H*_*o*_	*H_e(Ce)_*	*H_e(Cd)_*	*PIC*	*F*_*IS*_	*h*	*H*_*d*_	*π*	*D*	*F*
YAH	33	9.25	2.763	0.740	0.751	0.701	0.724	0.225**	6	0.67992	0.00491	0.96642	3.30335
JPH	41	10.50	2.821	0.770	0.815	0.760	0.793	0.103**	6	0.27317	0.00324	- 1.76785*	2.26913
MLW	40	9.167	2.496	0.657	0.726	0.678	0.698	0.262**	13	0.69231	0.00212	- 0.83499	- 6.79958*
WLW	65	11.75	2.734	0.740	0.820	0.765	0.801	0.275**	15	0.59712	0.00306	- 1.00333	- 1.80300

*N* number of individuals analyzed, *A* mean number of alleles per population, *A*_*i*_ allelic richness, *H*_*o*_ observed heterozygosity, *H*_*e* (*Ce*)_ expected heterozygosity under random chromosomal segregation, *H*_*e* (*Cd*)_ expected heterozygosity under random chromatid segregation, *PIC* polymorphic information content, *F*_*IS*_ inbreeding coefficients per population, *h* number of haplotypes, *H*_*d*_ haplotype diversity, *π* nucleotide diversity, *F* Fu’s (Fs) values, *D* Tajima’s (D) values

* *P* < 0.05

** *P* < 0.01.

After applying a sequential Bonferroni correction, the significant values of fixation indices were observed for almost half out of 48 locus-by-population comparisons (21 under random chromosomal segregation (*F*_*Cd*_) and 23 under random chromatid segregation (*F*_*Ce*_)), suggesting significant deviations from Hardy–Weinberg equilibrium (S2 Table). Under both random chromosomal segregation and random chromatid segregation, average values of *H*_*o*_ were lower than *H*_*e*_ in MLW, suggesting a deficit of heterozygosity. No significant linkage disequilibrium was detected at any of the 12 loci after Bonferroni correction (*P* > 0.05). Significant overall values of *F*_*IS*_ were obtained for four populations of *P*. *pingi* (*P <* 0.05), suggesting that inbreeding events may have occurred in the hatchery populations as well as in the two wild populations.

### Mitochondrial polymorphism

Sequence analysis of 791 bp of mtDNA control region revealed 45 variable sites, and the 179 sequences could be defined as 29 different haplotypes. H1 and H2 were the most common haplotypes, and recurred 36 and 92 times, respectively. Only one haplotype (H1) was shared among four populations. Five haplotypes were shared between JPH and WLW, while all other population pairs only shared two haplotypes. All the four populations had their own exclusive haplotypes. MLW and WLW both had the highest number of 9 private haplotypes. In contrast, the two hatchery populations had the lower private haplotype values of 3 in YAH and only 1 in JPH.

The overall haplotype diversity and nucleotide diversity was 0.69481 and 0.00507, respectively. MLW was characterized by the highest haplotype diversity (0.69231), yet the lowest nucleotide diversity (0.00212), suggesting that this population experienced a past rapid population expansion from an initial small effective population size [50].

### Genetic differentiation and population structure

AMOVA analyses revealed that most of the variances were found within populations (87.91% for microsatellite markers and 65.11% for mitochondrial control region). No significant group variability was detected by AMOVA analyses, with negligible variance Values of –3.54 for microsatellite markers (*F*_CT_ *=* 0.03543, *P* > 0.05) and –24.27 for mitochondrial control region (*F*_CT_ *= –* 0.24268, *P* > 0.05) (Table 2).

**Table 2:** Analysis of molecular variance (AMOVA) among four populations of *P*. *pingi*.

Source of variation	Microsatellite						mtDNA control region					
	d*f*	Sum of squares	Variance components	Variation(%)	Findex	*P*	d*f*	Sum of squares	Variance components	Variation(%)	Findex	*P*
Among group (%)	1	85.250	-0.34452 Va	-3.54	*F*_CT_ = -0.03543	>0.05	1	15.532	0.6138Va	-24.27	*F*_CT_ = -0.24268	>0.05
Among population (%)	2	278.911	1.52055 Vb	15.64	*F*_SC_ = 0.15100	<0.05	2	132.121	1.4964 Vb	59.16	*F*_SC_ = 0.47607	<0.05
Within population (%)	354	3026.395	8.54914 Vc	87.91	*F*_ST_ = 0.12093	<0.05	175	288.196	1.6468 Vc	65.11	*F*_ST_ = 0.34892	<0.05

*F*_*ST*_ values for microsatellite and *Φ*_*ST*_ values for mitochondrial data were significant for all population pairs (*P* < 0.05), with the highest *F*_*ST*_ value between YAH and JPH (0.196) and highest *ɸ*_*ST*_ value (0.653) between MLW and JPH. However, the differentiation between WLW and JPH was the lowest, despite its significant *P* value (*F*_*ST*_ *=* 0.026, *ɸ*_*ST*_ *=* 0.029). In addition, WLW and YAH showed relatively small genetic differentiation values of 0.161 for *F*_*ST*_ and 0.095 for *ɸ*_*ST*_ (Table 3).

**Table 3:** Pairwise genetic differentiation among the four populations of *P*. *pingi*, *ɸ*_*ST*_ from control region (below diagonal) and *F*_*ST*_ from microsatellites (above diagonal).

	YAH	JPH	MLW	WLW
YAH		0.196**	0.170**	0.161**
J PH	0.20978**		0.183**	0.026**
MLW	0.30063**	0.65377**		0.116**
WLW	0.09524*	0.02975*	0.55210**	

* *P* < 0.05

** *P* < 0.01.

**Fig 2:**
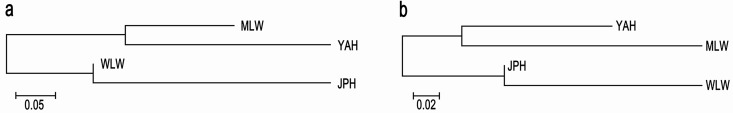
The neighbor-joining dendrogram of four populations of *P*. *pingi* based on microsatellite (a) and mtDNA control region (b).

The neighbor-joining dendrograms generated from microsatellite and mitochondrial data showed that YAH and MLW were clustered as one clade, while JPH and WLW were clustered as a separate clade (Fig 2).

**Fig 3:**
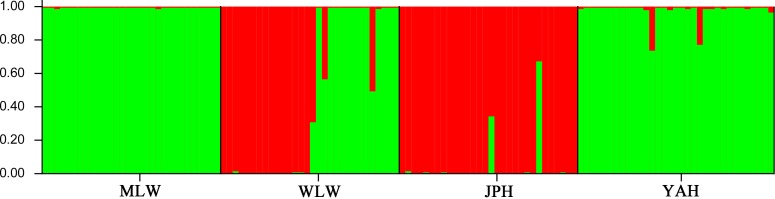
Results of Bayesian analysis (STRUCTURE) of *P*. *pingi* populations based on K = 2. Each column represents one individual and the colors represent the probability membership coefficient of that individual for each genetic cluster.

Bayesian analyses of population structure revealed a maximum ΔK value for two genetic clusters. The graphic representing this analysis showed two groups among the samples, one including MLW and YAH and another formed by JPH. WLW was apparently a mixture of these two groups (Fig 3).

**Fig 4:**
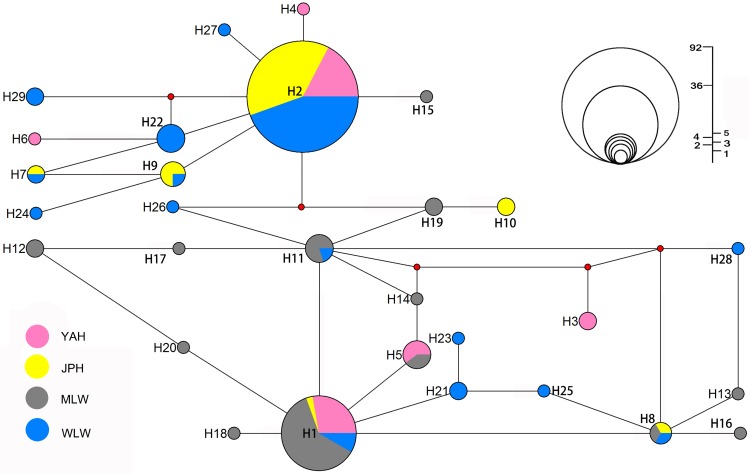
Haplotype network for 29 haplotypes of *P*. *pingi*, based on the median-joining algorithm. Circle sizes are proportional to haplotype frequency.

Additionally, the median-joining network clearly identified two main mtDNA haplogroups which centered on the two most common haplotypes (H1 and H2). Seventy samples from four populations clustered with one major haplogroup (1), while 109 individuals mainly from three different populations (YAH, JPH, and WLW) grouped with the largest haplogroup (2) (Fig 4).

### Demographic history

The results from BSP analysis showed that there was a population expansion for MLW and a bottleneck for JPH (S1 Fig). It was confirmed by negative and statistical significant values for Tajima’s D test in JPH (*Tajima's D = –*1.76785, *P =* 0.02400) and Fu’s FS test in MLW (*Fu's Fs = –* 4.70921, *P =* 0.02500). Moreover, bottleneck analysis did not detect any deviation from shift test mode in allele frequencies (all populations showed an L-shaped distribution of allele frequencies). However, a significant heterozygosity excess (*P =* 0.0302) was observed in JPH under the TPM model, suggesting recent reductions in effective population size in JPH.

### Equilibrium testing and historical gene flow

In 2MOD analysis, the probability of given model was estimated from the portions of 0 or 1 in the output file. And all interactions in both runs selected the migration–drift model (100% support).

Analysis from the coalescent-based method using the IM model showed that there were significant historical gene flows between the two wild populations. Immigration from MLW to WLW was slightly higher than from the opposite direction (1.8 vs. 1.4) (S2 Fig).

## Discussion

### Moderate genetic diversity within *P*. *pingi* populations

Genetic variation and evolutionary processes is highly important for the sustainability of many species [51]. A previous study suggested that the population resource of *P*. *pingi* in Yalong River was not rich and the population structure was simple [14]. In the present study, the level of microsatellite genetic diversity in the wild *P*. *pingi* (*H*_*o*_ *=* 0.657–0.740, *H*_*e*_(*c*_*e*_) *=* 0.726–0.820, *H*_*e*_(*c*_*d*_) *=* 0.678–0.765, *A =* 9.167–11.75) was much higher than that reported in 13 other freshwater fishes (*H*_*o*_ *=* 0.46, *H*_*e*_ *=* 0.54, *A =* 9.1) [52]. However, the mitochondrial data revealed slightly lower *H*_*d*_ values of 0.59712–0.69231 for wild *P*. *pingi* compared to that of 0.7118–0.8800 for some other teleostean fishes [53,54,55,56]. That microsatellites are more sensitive to detect genetic variability in species than the mtDNA control region may be the explanation of less concordance between the microsatellite data and mtDNA results [57]. Despite the relatively low *H*_*d*_ values observed in wild *P*. *pingi*, the levels of *π* detected within populations (*π =* 0.00212–0.00306) are very similar to that reported for other teleostean fishes (*π =* 0.00143–0.00280). We, therefore, suggest that the two wild populations of *P*. *pingi* in the two tributaries of the Yalong River showed a moderate genetic diversity, which was higher than previously reported by Yue et al. [14]. Nevertheless, since only two wild river populations were analyzed in this study, further investigation for other wild populations in the Yalong River drainage should be carried out to further confirm the present findings.

### Contrasting levels of genetic diversity between the two wild *P*. *pingi*: tributary as a refuge

Additionally, the genetic diversity of a species, combined with ecological and biological data is indispensable for clarifying the purpose and priority of conservation, and for the captive breeding programs [58]. In this study, the highest level of genetic diversity was found in the Woluo River population (WLW), however, the lowest level was observed in the Muli River population (MLW), which was even lower than YAH and JPH. In contrast to the studies that detected higher genetic diversity in wild fish compared to that of hatchery ones [18,29], our findings were somewhat unexpected. Freshwater fish populations are particularly susceptible to habitat degradation and to the introduction of exotic species [59]. Anthropogenic activity such as hydroelectric development has been the primary cause of habitat alteration in many rivers. Previous research has found that the fish assemblages in the middle and lower Yalong River have been dominated by lentic species instead of lotic species after the impoundment of the Ertan Reservoir [6]. Moreover, some small non-indigenous fishes have already threatened the native species [6]. Considering the circumstances in our study area, it seems that the contemporary state of preservation of the two tributaries in the lower Yalong River may account for the observed diametrically opposite scenarios in the genetic diversity between WLW and MLW. The Muli River is currently subjected to higher levels of anthropogenic impact (a combination of fishing pressure and habitat alteration due to hydropower construction) compared to the Woluo River.

In a previous study for fishes inhabiting main streams of the snowfall region in northern Honshu of Japan, tributaries might function as seasonal refuges and even as breeding habitat during the flood period [60]. In our study, native fish species, especially endemics, that preferred lotic environments in the Muli River, such as *P*. *pingi*, have become restricted to a few tributaries due to hydrological alteration and habitat fragmentation in the main channels [6]. Inward migration from main channel populations replenish the population of *P*. *pingi* in the Woluo River. Results of Bayesian analysis (STRUCTURE) confirmed it by showing that WLW also shares some of genetic component with MLW. In such cases, the Wuoluo River could serve as a refuge for *P*. *pingi* from anthropogenic disturbance. Additionally, the highest levels of genetic diversity observed in WLW would coincide with results revealed by some studies that tributaries may harbor a particular portion of the total genetic diversity of a species [27]. This was further evidenced by the presence of fifteen private microsatellite alleles and nine private mitochondrial haplotypes in WLW.

Based on these findings, management actions for *P*. *pingi* should be preferentially focused on maintaining the particular portions of genetic diversity in the tributaries with less anthropogenic impacts such as the Woluo River.

### Reduced intra-population genetic variation in hatchery population for release

Maintaining a high genetic variation in the hatchery samples is very critical for the success of the *P*. *pingi* captive breeding and hatchery release program. As for the two hatchery populations in our study, considerable genetic variations was observed for YAH, which reinforces result of our investigation that the parental fish of YAH were collected from multiple water systems, such as the Yalong, Qingyi, and Dadu rivers. We, therefore, suggest that abundant geographical sources of parents during its founding, subsequent mutation over generations (generating 9 private alleles) [61], or an artificial selection constitute the three major factors determining the levels of genetic diversity in YAH.

However, reduced intra-population genetic variation of JPH was detected by the mitochondrial control region dataset (*H*_*d*_ *=* 0.27317, *π =* 0.00324). As reported for other fishes [58], poorly breeding with small number of parents participating in reproduction, a recent bottleneck event together with subsequent occurrences of inbreeding (*P =* 0.0005) appeared to be the main explanations for the reductions of variabilities in JPH. Further massive releasing of hatchery fish with reduced genetic diversity might cause adverse impacts on adapted gene pools, consequently decreasing reproduction, growth, and adaption of wild populations [62]. To improve the quality of hatchery-released fish and the effectiveness of hatchery release as a conservation strategy of *P*. *pingi*, the use of appropriate sets of breeders for the establishment of founder broodstocks will be critical to maintain the genetic diversity in the hatchery fish.

### Similar patterns of microsatellite and mitochondrial genetic structure

Information on the population structure of fish species is fundamental to the establishment of Evolutionary Significant Units which is important to maintain genetic integrity [63]. In the present study, the two molecular markers revealed a similar pattern of genetic structure for the samples of *P*. *pingi* analyzed. There was no significant differences in population structure detected between hatchery and wild groups by AMOVA analysis. This result is congruent with the fact that the original *P*. *pingi* broodstocks of both hatcheries were completely or partly collected from the Yalong River system.

Moderate to high levels of differentiation were found between the hatchery populations and between the hatchery and wild populations (with exception for JPH and WLW). According to Wright [64], the range of *F*_*ST*_ values were divided into four layers by 0.05, 0.15, and 0.25 which correspond to four levels of genetic differentiation (weak, moderate, high and great). Regarding these, high genetic differentiation between YAH and JPH (*F*_*ST*_ *=* 0.196, *ɸ*_*ST*_ *=* 0.20978) was detected, further confirmed by their distinct genetic compositions revealed by structure analysis. This situation is probably related to different geographical sources of broodstocks during its founding and subsequent artificial selection of *P*. *pingi*.

While, weak genetic differentiation was found between JPH and WLW (*F*_*ST*_ *=* 0.026, *ɸ*_*ST*_ *=* 0.029). Low genetic divergence, and the results from the NJ dendrogram and Structure analysis indicated closer relationship between JPH and WLW, as confirmed by five mtDNA haplotypes commonly shared between them. It implies that the parental fish of *P*. *pingi* in the Fish Reproduction Station of the Jinping-Guandi might have originated from the Woluo River. Similarly, closer genetic relationship between the YAH and MLW was revealed by the NJ dendrogram and Bayesian clustering results (Fig 3), which suggests a possible geographical source of the *P*. *pingi* broodstocks in the Zhougonghe Yayu Company was from the Muli River. In fact, the parental fish of YAH were widely collected from the Yalong, Qingyi, and Dadu rivers. Since only the populations from one river was analyzed in the present study, further studies of this fish from other river systems (e.g., he Qingyi and Dadu rivers) should be carried out to confirm the geographical distributions of original broodstocks of YAH, although such analyses may be constrained by the challenge of sampling in these rivers.

The findings about genetic relations between hatchery and wild *P*. *pingi* may provide some practical guidance for conservation strategies such as hatchery release. Since releasing hatchery fish that are genetically divergent with the wild may cause disturbance to the population structure of the wild populations, it is reasonable to release hatchery individuals to river where their parental fish were first captured [65].

### Remarkable genetic differentiation between two wild *P*. *pingi* populations on a small geographic scale

In the present study, the wild *P*. *pingi* populations (MLW and WLW) from the geographically closer sampling locations showed evident genetic differences, although there was no apparent physical barrier restricting their connectivity. In addition, studies of wild populations in several fishes have revealed high genetic differences between main river channel populations and tributary populations [27,66,67]. This genetic isolation may have been caused by differences in habitat quality (e.g., physico-chemical properties, and river flow and hydrology) and human interference [68]. Some authors have argued that abrupt genetic divergence over small spatial distances might be caused by gene flow reduction among ancestral populations [69] and contemporary physical or temporal barriers [70,71]. However, the result of analyses on historical gene flow indicated that there were significant gene exchanges between MLW and WLW. The 2MOD analysis gave great support to a balance between migration and drift for the two wild populations in local demes, suggesting that contemporary population differentiation reflected on-going dispersal rather than historical isolation. Moreover, the considerable levels of genetic differentiation observed in natural populations may also be attributed to population fluctuations [72,73]. Taking into consideration of various elements, we therefore speculate that genetic differentiation between MLW and WLW may have been caused by different hydrogeological conditions (both natural and anthropogenic hydrological changes) between the two sampling locations as well as possible spatial population expansion of the MLW population.

Given the existence of the noticeable differences in the genetic structure between MLW and WLW, it seems that on-going gene flows between MLW and WLW were likely to have been very limited even within this small geographic distance. The two populations as well as other geographic populations of *P*. *pingi* are threatened by habitat fragmentation due to damming on the Yalong River, which in turn lead to geographic fragmentation of fish populations. Such populations may have experienced different natural selection regimes, and, as a result, are exhibit independent demographic processes and associated differences in their genetic diversity as well as significant genetic structure among these populations. Based on our findings, and until additional studies are performed at finer geographic scales, tributaries of major rivers probably provide the best basis for delineating possible expected increasing inter-populational differences in genetic structure within this species.

However, there is always a possibility that if such genetic flow continues, the genetic differentiation between these two wild populations will diminish. In consideration of the significant genetic divergence between MLW and WLW, caution should be taken when establishing conservation strategies for this fish. The Muli River Tributary has clearly suffered more hydrological changes than the Woluo Tributary. With reduced genetic diversity in MLW, the special proportion of genetic diversity maintained in this population might be lost. Moreover, the inbreeding events may occurred both in MLW and WLW as well, as indicated by *F*_*IS*_ analysis, which should be considered as an important factor to threat the persistence of wild populations. Consequently, we identified the two patches in the present study as two separate spatial scales that should be maintained to conserve the greatest possible genetic diversity of *P*. *pingi* within the basin. This means that conservation efforts should be directed to these two populations and their relevant habitats. Therefore, we suggest the creation of microscale protected areas for *P*. *pingi* in the Yalong River including stronger fishery supervision and habitat assessments in these areas. The creation of some protected areas is particularly important for the Woluo River Tributary, which likely functions as an important refuge for this species (and possibly for other lotic species as well). Protecting and maintaining the high genetic diversity observed in the Woluo Tributary may be critical for the future.

### Implications for Management

The presence of genetically differentiated populations indicates potential values for two ESUs and MUs as they represent independent evolutionary trajectories [73]. Pronounced levels of genetic diversity were found in the Woluo River population. This population also consists of two genetic groups, as revealed by Bayesian clustering analysis, which highlights the possible critical value of the Woluo Tributary for harboring important portions of genetic diversity of *P*. *pingi*. The Woluo Tributary has suffered less from anthropogenic disturbances than the surrounding areas. It is thus necessary to define Woluo River as a refuge which plays a central role in the persistence of *P*. *pingi* populations. In addition, WLW was found have relatively low genetic differentiation with YAH and JPH. The Woluo Tributary within the Yalong River system is a conservation priority for *P*. *pingi*. In addition, with regard to the reduced genetic diversity of the Muli River population, we argue the critical importance of enhancement and releasing measures in the Muli Triburary to increase the effective population size and genetic variation of these local populations as represented by MLW.

Previous studies elsewhere have demonstrated that small effective broodstock size for the production and releases of hatchery-reared fish is the primary cause of genetic variation reductions along with increased inbreeding [17,19]. Such negative effects were explicitly observed in JPH. Thus we suggest a focused effort be undertaken to document and increase the effective number of breeders in the future research, as well as a parentage analysis. We observed that YAH and JPH consisted of different genetic compositions. Consequently, selective breeding is suggested for these two hatcheries, to benefit genetic properties of released fish, thus preserve the genetic diversity necessary for continued and sustainable development of *P*. *pingi* resource in Yalong River.

## Supporting Information

S1 Fig Bayesian skyline plot of mitochondrial lineages of four populations of *P*. *pingi*. (TIF)

S2 Fig Estimates of historical gene flow (m) in the two wild *P*. *pingi* populations. Boxes represent sampled and ancestral populations, horizontal lines represent splitting times, curved arrows represent migration and numbers above or below arrows represent migration rates in the direction of the arrow. Time is represented as depth on the vertical axis, with the sampled population names at the top of the figure at the most recent time point. *P < 0.05.
(TIF)

S3 Fig The copy of the approval for conducting our experiments from the Sichuan Municipal Bureau of Aquatic Products. (TIF)

S1 Table Twelve microsatellite loci used in population genetic diversity and structure analysis of *P*. *pingi*. (DOCX)

S2 Table Genetic variation of *P*. *pingi* at twelve microsatellite loci. (DOC)
