# Supplementary figures and images for: Genetic Diversity and Structure Analysis of Percocypris pingi (Cypriniformes: Cyprinidae): Implications for Conservation and Hatchery Release in the Yalong River
Source: PLoS One. 2016 Dec 2;11(12):e0166769. doi: 10.1371/journal.pone.0166769 (PMC5135059; doi:10.1371/journal.pone.0166769)

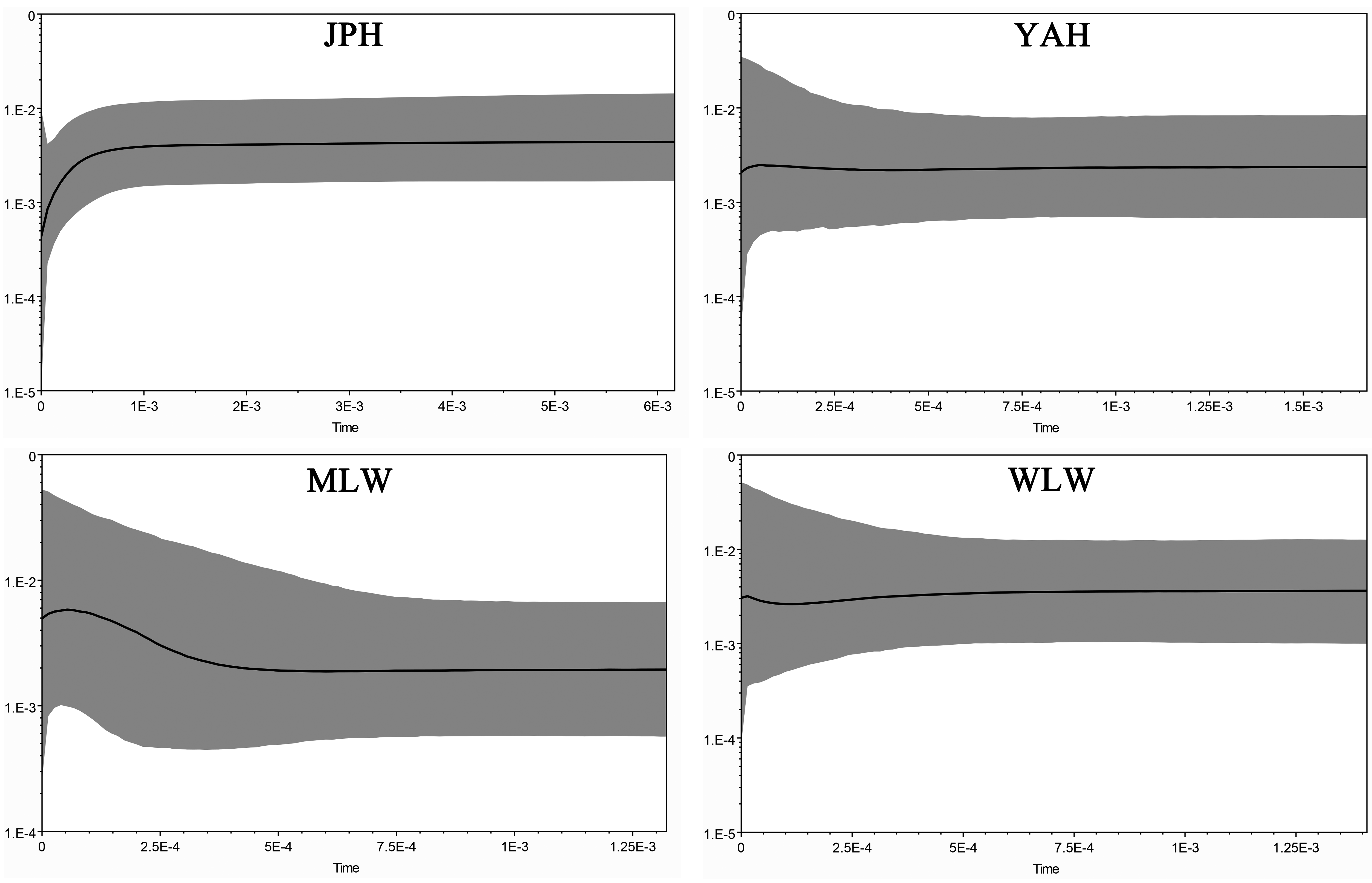

Supplement: S1 Fig — (TIF) [file pone.0166769.s001.tif]

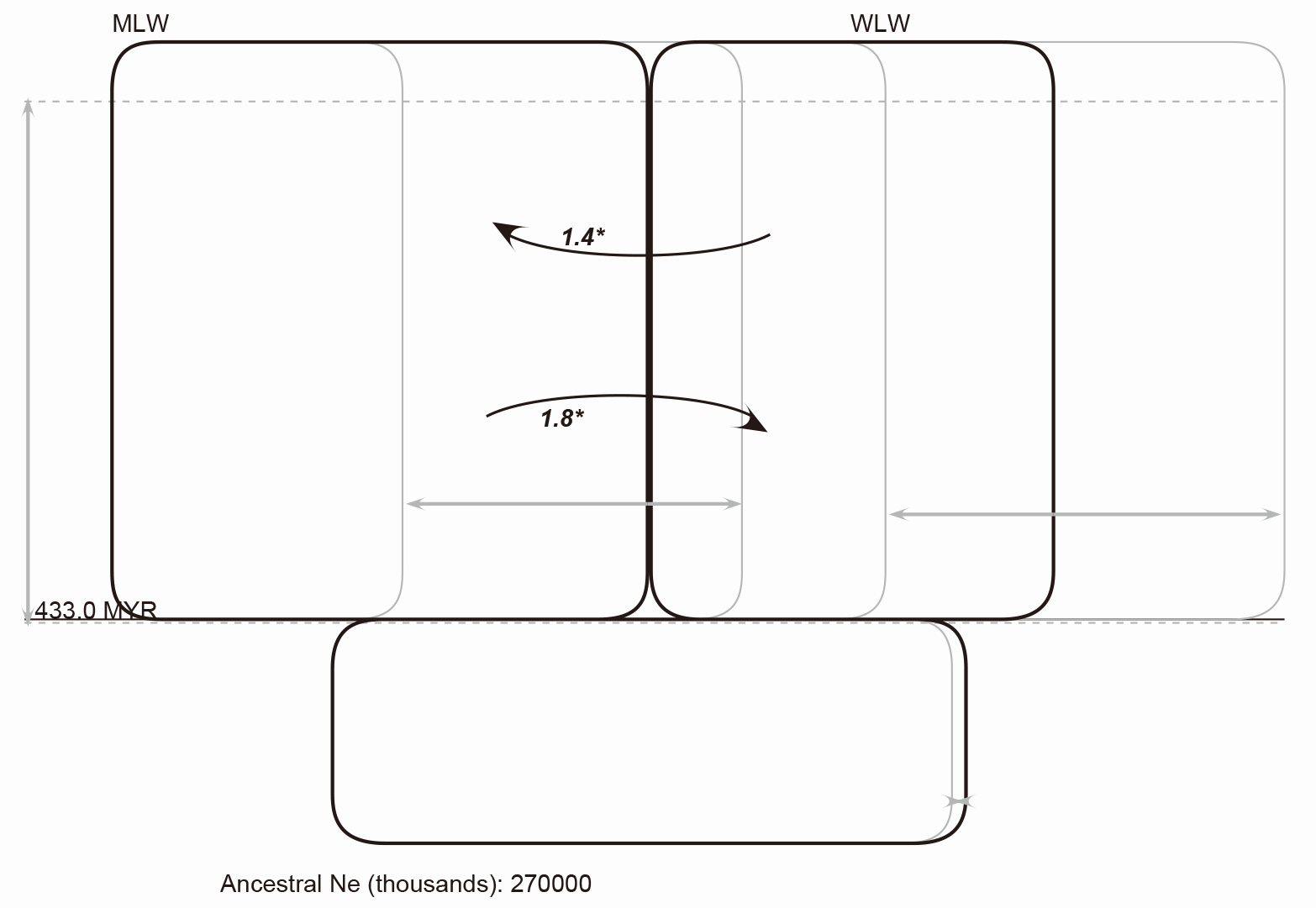

Supplement: S2 Fig — Boxes represent sampled and ancestral populations, horizontal lines represent splitting times, curved arrows represent migration and numbers above or below arrows represent migration rates in the direction of the arrow. Time is represented as depth on the vertical axis, with the sampled population names at the top of the figure at the most recent time point. *P < 0.05. (TIF) [file pone.0166769.s002.tif]

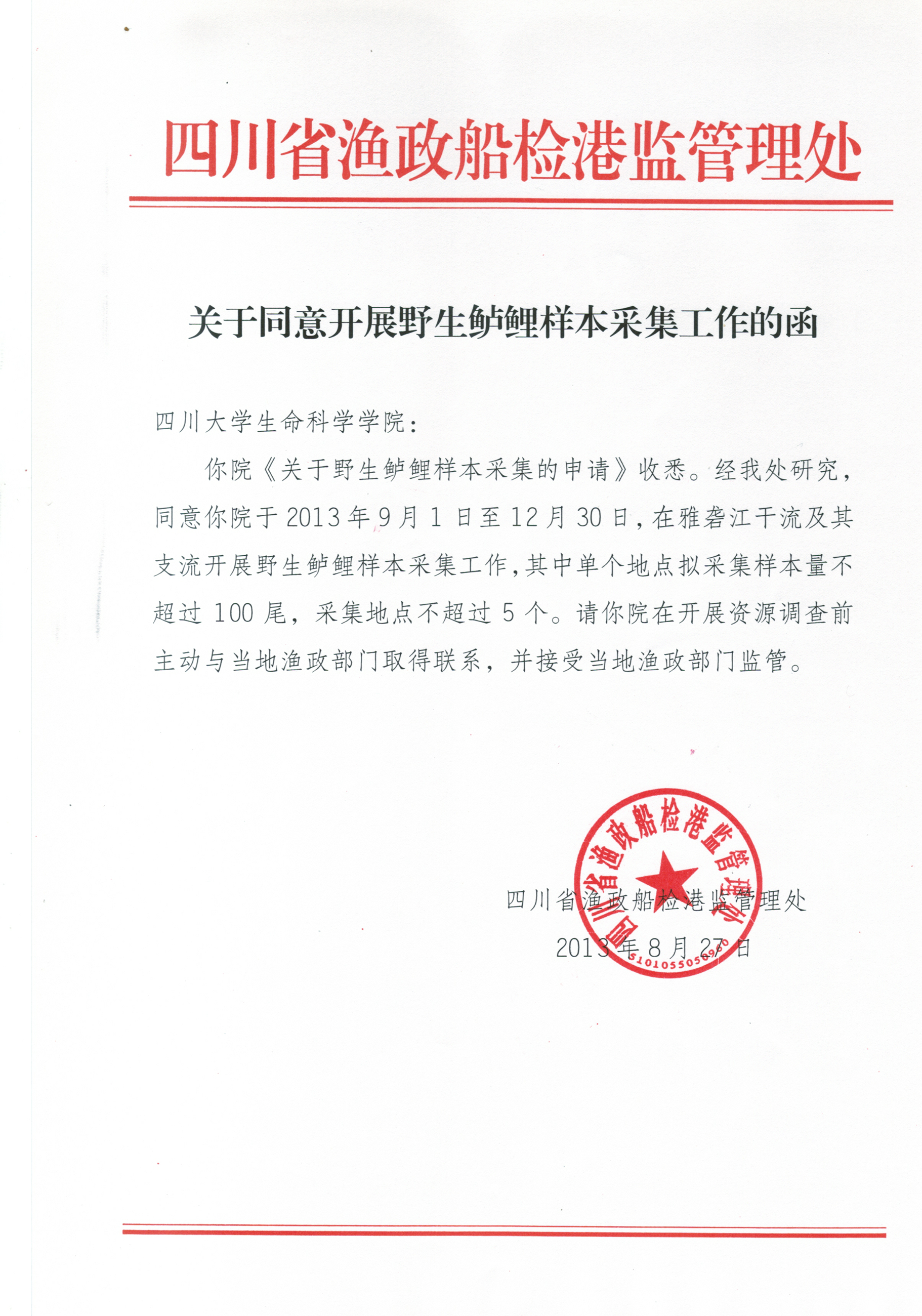

Supplement: S3 Fig — (TIF) [file pone.0166769.s003.tif]
