# Supplementary material for: Genetic Diversity and Structure Analysis of Percocypris pingi (Cypriniformes: Cyprinidae): Implications for Conservation and Hatchery Release in the Yalong River
Source: PLoS One. 2016 Dec 2;11(12):e0166769. doi: 10.1371/journal.pone.0166769 (PMC5135059; doi:10.1371/journal.pone.0166769)
Supplement: S1 Table — (DOCX) [file pone.0166769.s004.docx]

| **S1 Table.** **Twelve microsatellite loci used in population genetic diversity and structure analysis of *P. pingi*.** | | | | |
| --- | --- | --- | --- | --- |
| **Locus/accession no.** | **Primer sequence (5’-3’)** | **Repeat motif** | **Tm(°C)** | **Reference** |
| PM10/KJ473456 | F: TCCAGAAAGCTTGGAAAGAAA  R: TGTGAACCAGGGTTATGCAA | (AGAT)11 | 64°C | (Deng 2015) |
| PM17/KJ473458 | F: TTTCTGGTTGTGACAGTTAAAGGA  R: GGCATCTACACCAATGAAGGA | (TATC)17 | 60°C | (Deng 2015) |
| PM49/KJ473459 | F: AAGCCTGTTTTTGCCTCAGA  R: GGGAAGTTAGCAAGGGAAGG | (TAAA)11 | 62°C | (Deng et al. 2015) |
| PM32/KJ473463 | F: AAGAAAGGGTTGCATGTGGT  R: TGCCTTTGTATGGGACCTCT | (ATCT)10 | 64°C | (Deng et al. 2015) |
| PM16/KJ473466 | F: TGTCCTGTCCTGTTGCTGTC  R: GTGGAATAAGGATGCGGTCT | (GATA)15 | 64°C | (Deng 2015) |
| PM08/KJ473467 | F: CTTGCTTGGCTTGCAGTTTT  R: TGAATGGTGGTGTCTTGTGG | (TATC)10 | 61°C | (Deng 2015) |
| PM25/KJ473470 | F: GTTAACCTGGAGGGCAAACA  R: CTGGATGCATACTTGTCCGTA | (TCTT)13 | 61°C | (Deng et al. 2015) |
| PM40/KJ473471 | F: GGGACGGTTGTCAAAGAAGA  R: GGAACGACATGAGGGTGAGT | (TTTG)10 | 62°C | (Deng 2015) |
| PM23/KJ473474 | F: AACCAGCAAACCAGACCAAC  R: CACATCACCTTCAGCAGCTC | (ATCT)21 | 64°C | (Deng et al. 2015) |
| PM20/KJ473475 | F: TTTTGAAAATTGACATCCAGAA  R: TCACATGCACATTTCCAATACA | (CTAT)12 | 60°C | (Deng et al. 2015) |
| PM19/KJ473478 | F: GTTGGCTTGAGGAAGCCTTA  R: CATTTACCTCACACCTCTGAACTC | (TAGA)21 | 64°C | (Deng et al. 2015) |
| PM15/KJ473481 | F:AGATAGAAGGAATAATTCACCCAATA  R: AGCAGCAGAACGACAGATAGA | (TCTA)16 | 64°C | (Deng et al. 2015) |
