# Supplementary material for: Genetic Diversity and Structure Analysis of Percocypris pingi (Cypriniformes: Cyprinidae): Implications for Conservation and Hatchery Release in the Yalong River
Source: PLoS One. 2016 Dec 2;11(12):e0166769. doi: 10.1371/journal.pone.0166769 (PMC5135059; doi:10.1371/journal.pone.0166769)
Supplement: S2 Table — (DOC) [file pone.0166769.s005.doc]

**S2 Table. Genetic variation of *P. pingi* at twelve microsatellite loci.**

| ***Population*** | **Microsatellite locus** | | | | | | | | | | | | Means |
| --- | --- | --- | --- | --- | --- | --- | --- | --- | --- | --- | --- | --- | --- |
|  | PM15 | PM10 | PM23 | PM32 | PM40 | PM49 | PM16 | PM25 | PM08 | PM17 | PM19 | PM20 |  |
| **YAH** | | | | | | | | | | | | | |
| *N* | 33 | 33 | 33 | 33 | 33 | 33 | 33 | 33 | 33 | 33 | 33 | 33 | 33 |
| *A* | 9 | 7 | 12 | 10 | 7 | 8 | 11 | 9 | 10 | 7 | 10 | 11 | 9.25 |
| *Ai* | 2.818 | 2 | 3.515 | 2.97 | 2.273 | 2.606 | 2.939 | 2.667 | 2.848 | 2.636 | 3.061 | 2.818 | 2.731 |
| *H_o_* | 0.702 | 0.606 | 0.919 | 0.798 | 0.566 | 0.727 | 0.783 | 0.753 | 0.758 | 0.687 | 0.818 | 0.763 | 0.733 |
| *H_e_*(*_Ce_*) | 0.76 | 0.694 | 0.872 | 0.756 | 0.513 | 0.757 | 0.824 | 0.787 | 0.843 | 0.649 | 0.828 | 0.729 | 0.751 |
| *H_e_*(*_Cd_*) | 0.709 | 0.647 | 0.814 | 0.705 | 0.479 | 0.706 | 0.769 | 0.735 | 0.787 | 0.606 | 0.773 | 0.68 | 0.701 |
| *F*(*_Ce_*) | 0.076***** | 0.126***** | -0.054 | -0.056 | -0.102 | 0.039***** | 0.05 | 0.044***** | 0.102 | -0.058 | 0.012 | -0.047 | 0.015 |
| *F*(*_Cd_*) | 0.010***** | 0.064***** | -0.13***** | -0.131***** | -0.181***** | -0.03 | -0.018 | -0.024 | 0.037 | -0.134***** | -0.059 | -0.121***** | -0.056 |
| *PIC* | 0.728 | 0.646 | 0.860 | 0.725 | 0.493 | 0.731 | 0.808 | 0.755 | 0.824 | 0.616 | 0.808 | 0.691 | 0.724 |
| **JPH** | | | | | | | | | | | | | |
| *N* | 41 | 41 | 41 | 41 | 41 | 41 | 41 | 41 | 41 | 41 | 41 | 41 | 41 |
| *A* | 13 | 8 | 14 | 10 | 7 | 7 | 13 | 11 | 12 | 9 | 13 | 9 | 10.5 |
| *Ai* | 3.463 | 2.268 | 3.098 | 2.415 | 2.927 | 2.829 | 2.829 | 2.805 | 2.732 | 2.878 | 2.683 | 2.927 | 2.821 |
| *H_o_* | 0.911 | 0.683 | 0.825 | 0.654 | 0.797 | 0.772 | 0.78 | 0.772 | 0.724 | 0.74 | 0.772 | 0.813 | 0.77 |
| *H_e_*(*_Ce_*) | 0.87 | 0.83 | 0.885 | 0.721 | 0.779 | 0.781 | 0.816 | 0.848 | 0.854 | 0.767 | 0.813 | 0.811 | 0.815 |
| *H_e_*(*_Cd_*) | 0.812 | 0.775 | 0.826 | 0.673 | 0.727 | 0.729 | 0.762 | 0.791 | 0.797 | 0.716 | 0.759 | 0.757 | 0.76 |
| *F*(*_Ce_*) | -0.047 | 0.177***** | 0.067***** | 0.093 | -0.023 | 0.011 | 0.044***** | 0.089***** | 0.152***** | 0.035***** | 0.05***** | -0.002***** | 0.054 |
| *F*(*_Cd_*) | -0.121***** | 0.119***** | 0.001 | 0.028 | -0.096 | -0.059 | -0.025 | 0.024 | 0.092 | -0.033***** | -0.018***** | -0.073 | -0.013 |
| PIC | 0.856 | 0.809 | 0.874 | 0.692 | 0.745 | 0.748 | 0.800 | 0.830 | 0.837 | 0.742 | 0.791 | 0.787 | 0.793 |
| **MLW** | | | | | | | | | | | | | |
| *N* | 40 | 40 | 40 | 40 | 40 | 40 | 40 | 40 | 40 | 39 | 40 | 40 | 39.917 |
| *A* | 11 | 10 | 7 | 10 | 8 | 6 | 7 | 11 | 7 | 12 | 13 | 8 | 9.167 |
| *Ai* | 3.325 | 2.425 | 2.675 | 2.7 | 2.425 | 2.775 | 1.95 | 2.475 | 1.925 | 1.872 | 2.875 | 2.525 | 2.496 |
| *H_o_* | 0.85 | 0.717 | 0.725 | 0.733 | 0.687 | 0.742 | 0.579 | 0.671 | 0.45 | 0.359 | 0.721 | 0.65 | 0.657 |
| *H_e_*(*_Ce_*) | 0.868 | 0.815 | 0.777 | 0.784 | 0.661 | 0.69 | 0.734 | 0.73 | 0.693 | 0.437 | 0.871 | 0.657 | 0.726 |
| *H_e_*(*_Cd_*) | 0.81 | 0.761 | 0.726 | 0.732 | 0.617 | 0.644 | 0.685 | 0.681 | 0.647 | 0.407 | 0.813 | 0.613 | 0.678 |
| *F*(*_Ce_*) | 0.02 | 0.12***** | 0.067 | 0.064 | -0.041***** | -0.075 | 0.211***** | 0.081 | 0.351***** | 0.178***** | 0.172***** | 0.011 | 0.095 |
| *F*(*_Cd_*) | -0.05***** | 0.058***** | 0.001 | -0.002 | -0.115***** | -0.152 | 0.155***** | 0.016 | 0.305***** | 0.119***** | 0.113***** | -0.06***** | 0.031 |
| *PIC* | 0.854 | 0.790 | 0.741 | 0.764 | 0.627 | 0.652 | 0.691 | 0.690 | 0.650 | 0.425 | 0.859 | 0.628 | 0.698 |
| **WLW** | | | | | | | | | | | | | |
| *N* | 64 | 65 | 65 | 65 | 65 | 65 | 65 | 63 | 65 | 65 | 65 | 65 | 64.75 |
| *A* | 14 | 8 | 13 | 15 | 10 | 8 | 13 | 12 | 11 | 12 | 14 | 11 | 11.75 |
| *Ai* | 3.359 | 2.569 | 2.938 | 2.308 | 2.615 | 2.831 | 2.415 | 2.921 | 2.692 | 2.738 | 2.908 | 2.508 | 2.734 |
| *H_o_* | 0.891 | 0.731 | 0.805 | 0.551 | 0.756 | 0.792 | 0.633 | 0.77 | 0.731 | 0.71 | 0.813 | 0.7 | 0.74 |
| *H_e_*(*_Ce_*) | 0.878 | 0.787 | 0.892 | 0.651 | 0.803 | 0.809 | 0.843 | 0.869 | 0.868 | 0.813 | 0.851 | 0.776 | 0.82 |
| *H_e_*(*_Cd_*) | 0.82 | 0.735 | 0.832 | 0.607 | 0.75 | 0.755 | 0.787 | 0.811 | 0.811 | 0.759 | 0.795 | 0.724 | 0.765 |
| *F*(*_Ce_*) | -0.014 | 0.072***** | 0.097 | 0.153***** | 0.058 | 0.02 | 0.249***** | 0.114 | 0.159***** | 0.126***** | 0.045 | 0.098 | 0.097 |
| *F*(*_Cd_*) | -0.087***** | 0.006***** | 0.033 | 0.092 | -0.009 | -0.05 | 0.195 | 0.05 | 0.098 | 0.064 | -0.023 | 0.033 | 0.033 |
| *PIC* | 0.866 | 0.759 | 0.882 | 0.637 | 0.779 | 0.784 | 0.826 | 0.855 | 0.855 | 0.790 | 0.835 | 0.745 | 0.801 |
| **Total** |  |  |  |  |  |  |  |  |  |  |  |  |  |
| *N* | 179 | 179 | 179 | 179 | 179 | 179 | 177 | 179 | 178 | 179 | 179 | 178 | 178.66 |
| *A* | 11 | 14 | 15 | 11 | 9 | 13 | 14 | 13 | 14 | 16 | 13 | 14 | 13.083 |
| *Ai* | 2.514 | 2.743 | 2.642 | 2.709 | 2.715 | 2.441 | 2.797 | 2.525 | 2.601 | 2.799 | 2.709 | 3.275 | 2.706 |
| *H_o_* | 0.711 | 0.755 | 0.683 | 0.758 | 0.735 | 0.672 | 0.750 | 0.670 | 0.649 | 0.760 | 0.736 | 0.862 | 0.729 |
| *H_e_*(*_Ce_*) | 0.847 | 0.882 | 0.770 | 0.822 | 0.785 | 0.873 | 0.858 | 0.873 | 0.827 | 0.877 | 0.857 | 0.883 | 0.846 |
| *H_e_*(*_Cd_*) | 0.791 | 0.823 | 0.719 | 0.767 | 0.733 | 0.815 | 0.801 | 0.815 | 0.772 | 0.819 | 0.800 | 0.824 | 0.790 |
| *F*(*_Ce_*) | 0.160***** | 0.144***** | 0.112 | 0.078***** | 0.065***** | 0.230***** | 0.125***** | 0.232***** | 0.215***** | 0.134***** | 0.141***** | 0.023***** | 0.139 |
| *F*(*_Cd_*) | 0.100***** | 0.083***** | 0.049 | 0.012***** | -0.002 | 0.175***** | 0.063***** | 0.177***** | 0.159 | 0.072***** | 0.079***** | -0.047***** | 0.078 |
| *PIC* | 0.828 | 0.872 | 0.757 | 0.801 | 0.760 | 0.861 | 0.844 | 0.861 | 0.810 | 0.865 | 0.842 | 0.872 | 0.831 |

*N* number of individuals sampled, *A* mean number of alleles per population, *A_i_* allelic richness, *H_o_* observed heterozygosity, *H_e_*(*_Ce_*) expected heterozygosity under random chromosomal segregation, *H_e_*(*_Cd_*) expected heterozygosity under random chromatid segregation, *PIC* polymorphic information content, *F*(*_Ce_*) fixation coefficient under random chromosomal segregation, *F*(*_Cd_*) fixation coefficient under random chromatid segregation, * significant fixation coefficient.
